# Towards an Analytical Age-Dependent Model of Contrast Sensitivity Functions for an Ageing Society

**DOI:** 10.1155/2015/625034

**Published:** 2015-05-20

**Authors:** Karine Joulan, Roland Brémond, Nicolas Hautière

**Affiliations:** ^1^COSYS, IFSTTAR, Université Paris-Est, 77447 Marne-la-Vallée, France; ^2^Continental Automotive France SAS, 1 avenue Paul Ourliac, 31036 Toulouse, France

## Abstract

The Contrast Sensitivity Function (CSF) describes how the visibility of a grating depends on the stimulus spatial frequency. Many published CSF data have demonstrated that contrast sensitivity declines with age. However, an age-dependent analytical model of the CSF is not available to date. In this paper, we propose such an analytical CSF model based on visual mechanisms, taking into account the age factor. To this end, we have extended an existing model from Barten (1999), taking into account the dependencies of this model's optical and physiological parameters on age. Age-dependent models of the cones and ganglion cells densities, the optical and neural MTF, and optical and neural noise are proposed, based on published data. The proposed age-dependent CSF is finally tested against available experimental data, with fair results. Such an age-dependent model may be beneficial when designing real-time age-dependent image coding and display applications.

## 1. Introduction

Population ageing, defined as a process which increases the proportion of old people within the total population, is likely to become one of the main issues of our modern societies.

Among the numerous challenges, the visual performance in everyday tasks is impaired for older people compared to young ones, either in domestic, working, or driving tasks [1–4]. The dependence of visual performance on age has been intensively studied in order to identify and understand the underlying mechanisms which contribute to this slow degradation. Specifically, spatial and temporal contrast sensitivities have been studied in both photopic and scotopic conditions.

Outside of the vision science community, the Contrast Sensitivity Function (CSF) has become popular in computer vision [5–8], and taking the Human Visual System (HVS) into account is among the main design constraints in any kind of displays [9]. Because images are displayed for people who are likely to watch them, image coding also takes into account (and take advantage of) the HVS limited capacity [10, 11].

With the progress in image coding and processing hardware, it becomes possible to tune in real time the coding parameters as a function of the receiver's age: in videoconference tools or some smartphone applications, the system may be tuned to optimize the communication bandwidth [12, 13]; for these applications, the temporal CSF may help the designer as well as the spatial CSF. In terms of the visual displays, it would also be possible to enhance the image contrast with respect to the actual age of the observers [14]. In another industrial field, one may imagine that, in the near future, automotive lighting should be tuned to some driver's individual characteristics, such as age, in order to guarantee some level of visual performance [15, 16].

In this aim, it is necessary to have at disposal an analytical age-dependent model of CSF. Unfortunately, to our best knowledge, such a model does not exist. Since CSF data are nevertheless available at different ages, this paper aims at building such a CSF model upon them.

In the following, we first review the prior works, which are dealing with the decline of CSF with age. Then, we extend Barten's analytical model of the CSF [17], which is based on vision mechanisms, in order to propose an age-dependent CSF. In Section 3, we describe Barten's model and focus in Section 4 on the optical and neural factors which can possibly contribute to the contrast sensitivity decline with age. In Sections 5 and 6, we propose an age-dependent CSF model with the same structure as Barten's age-independent model. Finally, in Section 7, we compare the proposed model with contrast sensitivity data from the vision science literature.

## 2. Prior Works

Many authors have proposed CSF data for different ages [18–22]. Comparing the visual performance of young and old observers looking at static sine wave gratings, the CSF was found to decline with age for high spatial frequencies in the photopic domain and softly or not at all for lower frequencies.

The age effect is clearly different in photopic and mesopic/scotopic conditions, but the available data depend on the experimental conditions and on the observer's characteristics (e.g., visual pathologies), which were not always carefully controlled in past studies. According to Owsley's recent literature review [23], the main explanation of the CSF decline with age in photopic condition comes from optical factors.

First, the pupil aperture is smaller for older observers compared to young ones [24], which lowers the retinal illuminance [25]. The influence of the pupil diameter on visual acuity has been demonstrated long ago under various adaptation luminances [26]. The effect of the diminution of the pupil's diameter with ageing (senile miosis) is twofold: on the one hand, with a small diameter the retina is not illuminated enough, while on the other hand, it also increases the optical noise.

More factors contribute to the contrast sensitivity decline. The density of proteins in the crystallin lens increases with age, leading to an increase in intraocular diffusion [27–29]. Also, chromatic and geometric abberations are more important in the elderly who cannot accommodate sufficiently, due to the stiffening of the lens matter.

The first models simulating light scattering inside the eye used rough models of the crystallin and cornea's shapes and refractive index [30, 31]. The GRIN lens model allowed a better accuracy of the crystallin's shape [32]. Then, Liou and Brennan improved Blaker's model [33] and collected new data about the crystallin's deformations with age and accommodation. The lens was modeled as a conic function, and the radii for the anterior and posterior faces and the gradient refractive index were taken into account [34]. A model of the scattering and diffraction by proteins in the crystallin lens was proposed (MLB for Multilamellar Bodies); the proteins are simulated as spherical particles with a refractive index, an obscuration area, and a range of diameters [35]. Mie equations were proposed to describe light scattering in the eye medium [36, 37], which also occurs in the retina, in the iris, and in the sclera.

The “photon noise” describes the statistical fluctuations in the number of incident photons absorbed by the photoreceptors. The ratio of incident photon exciting these photoreceptors, called* quantum efficiency* [38], lowers with age, which contributes to lower the optical Modulation Transfer Function (MTF) of ageing people [18, 39].

Even if a lot of studies showed that optical factors are mainly responsible for the contrast sensitivity decline, Elliott et al. suggested that the diminution of the contrast sensitivity may also be due to neural mechanisms [40]. However, their experiment only manipulated the pupil's diameter and monochromatic aberrations. Other authors have studied neural age-related factors in contrast sensitivity [41, 42], suggesting that neural cells properties in the postretinal visual pathway may also explain the diminution of the contrast sensitivity through adulthood; no consensus has emerged so far, however. It was proposed for instance that the LGN cell's receptive field could enlarge with ageing [43], but alternative explanations were also proposed [41].

In the mesopic and scotopic ranges, in addition to optical factors, neural factors are important to understand the CSF decline with age. Whatever the experimental conditions, the magnitude of contrast sensitivity has been found nearly three times lower in old than in young observers. The decline is not uniform but appears at all spatial frequencies, which may be due to a loss of rod photoreceptor and ganglion cells [44, 45]. Rods degeneration, lower density, and dysfunction in aged observers have been suggested [44, 46]. Further studies are needed for a better understanding of neural and cortical mechanisms in scotopic conditions.

The latency of the rhodopsin increases for the elderly, leading to a slower dark adaptation of aged people [22], which contributes to their loss of contrast sensitivity at low luminance. Also, the retinal pigment epithelium and the Bruch membrane are thicker in older people, allowing more scattering of vitamin A, which in turn contributes to the regeneration cycle of the rhodopsin [22].

## 3. Reference Model

The Contrast Sensitivity Function describes the sensitivity of the human eye to sine wave gratings, which can be displayed at various spatial and temporal frequencies. Given sinusoidal luminance grating *L*(*x*, *y*, *t*), with mean luminance 〈*L*〉 (with *x* and *y* being the spatial dimensions and *t* being the temporal dimension), the noise spectral density Φ_*n*_ is related to the Fourier transform of *L* − 〈*L*〉, *F*:

(1) $$\begin{array}{c} \Phi_{n}\left(\;u,v,w\;\right)=\frac{1}{XYT}\frac{{\left|F\left(u,v,w\right)\right|}^{2}}{{\langle L\rangle }^{2}}, \end{array}$$

where *X*, *Y*, and *T* are maximum values in the spatial and temporal dimensions.

The contrast sensitivity of the eye is the inverse of the contrast threshold *m*_*t*_, above which a human observer can see a grating. This threshold depends on the modulation of internal noise into the eye *m*_*n*_. According to Barten [17], the probability density distribution of *m*_*n*_ is equal to

(2) $$\begin{array}{c} m_{n}\left(\;u,v,w\;\right)=2\sqrt{\frac{\Phi_{n}\left(u,v,w\right)}{XYT}}. \end{array}$$

Barten splits the spectral density of the internal noise Φ_*n*_ into two components. The input signal is filtered by the photon noise Φ_opt_ in the ocular media then by neural noise Φ_neu_. The former is modulated by lateral inhibition term *M*_lat_, so that (1) can be rewritten:

(3) $$\begin{array}{c} \Phi_{n}\left(\;u\;\right)=\Phi_{\text{opt}}M_{\text{lat}}^{2}\left(\;u\;\right)+\Phi_{\text{neu}}. \end{array}$$

Barten proposed that *m*_*t*_ is proportional to the probability density distribution of *m*_*n*_, after convolutions by the optical MTF (*M*_opt_) and by the lateral inhibition MTF (*M*_lat_) [17], which results in

(4) $$\begin{array}{c} m_{t}M_{\text{opt}}\left(\;u\;\right)M_{\text{lat}}\left(\;u\;\right)=km_{n}. \end{array}$$

According to Barten, *k* is the signal-noise ratio. Finally, Barten's CSF model is expressed as

(5) $$\begin{array}{c} \text{CS}{\text{F}}_{B}\left(\;u\;\right)=\frac{1}{m_{t}}=\frac{M_{\text{opt}}\left(u\right)}{2k}\frac{\sqrt{XYT}}{\sqrt{\Phi_{\text{opt}}+\left(\Phi_{\text{neu}}/M_{\text{lat}}^{2}\left(u\right)\right)}}. \end{array}$$

Among the different components of the CSF, some are age-dependent and will be discussed in more detail in the following. Then, an age-dependent CSF model based on (5) will be proposed, with explicit age-dependent factors. When experimental data is available, we have directly fitted these age-dependent factors in order to propose an analytical age-dependent function (Section 5). Since it was not always possible to model all the factors, the remaining ones were fitted with experimental CSF data with a Lagrangian optimization method (Section 6).

## 4. Age-Dependent Factors in Barten's Model

From (5), four main factors emerge: the optical and neural MTF (*M*_opt_ and *M*_lat_) and the optical and neural noises (Φ_opt_ and Φ_neu_). Age may impact all four factors.

*T* is the minimum between the eye's integration time *T*_*e*_ and the stimulus presentation time *T*_*o*_. In photopic condition and whatever the observer's age, *T*_*e*_ is roughly constant around 0.1 sec, but because the recovering of rhodopsin in aged observers is slower, one can expect higher values of *T*_*e*_ in mesopic and scotopic conditions for the elderly.

### 4.1. Optical MTF

The optical MTF describes the behavior of the input signal passing through the optical elements of the eye. Diffusion, the nature of the crystallin, and the pupil diameter lower the signal reaching the retina. These factors can be considered as low pass filters, and *M*_opt_ can be expressed as

(6) $$\begin{array}{c} M_{\text{opt}}\left(\;u\;\right)=\exp\left[\;-2\pi^{2}\sigma_{\text{opt}}^{2}{\left(\;\frac{u}{u_{\text{opt}}}\;\right)}^{2}\;\right]. \end{array}$$

Here, Barten's standard deviation *σ* has been split into two terms, *σ*_opt_ and *u*_opt_, making their counterpart easier to interpret: *σ*_opt_ is a dimensionless standard deviation of the optical MTF and *u*_opt_ is the cut-off frequency of the optical system (the eye), expressed in cpd.

#### 4.1.1. Optical Cut-Off Frequency

Unexpectedly, *u*_opt_ is not considered in Barten's model. Reference values are available in the vision science literature (e.g., *u*_opt_ = 40 cpd for *d* = 5 mm [47]), but no data was available for this parameter as a function of age. Thus, it was decided to estimate its sensitivity to age by fitting the available CSF data (see Section 6).

#### 4.1.2. Standard Deviation

According to Barten, the optical SD *σ*_opt_ depends on three terms:

(7) $$\begin{array}{c} \frac{\sigma_{\text{opt}}}{u_{\text{opt}}}=\sqrt{\sigma_{0}^{2}+d^{2}C_{ab}^{2}} \end{array}$$

*C*_*ab*_ links the increase of *σ*_opt_ with the pupil diameter *d*, which decreases with age. In the absence of data about the dependence of *C*_*ab*_ on age, we follow Barten and set *C*_*ab*_ = 0.08. Hopefully, Watson recently proposed a detailed model of the pupil diameter as a function of age [24] (see below Section 4.1.3).

Barten uses *σ*_0_ = 0.5 arcmin. It is the maximum of cycle per degree that a human eye can perceive (with this value, one can perceive as much as 60 cycles per degree in foveal vision). However, due to the lack of available age-dependent data, we estimated *σ*_0_ based on available CSF data as a function of age (see Section 6).

#### 4.1.3. Pupil Diameter

The higher the pupil diameter, the higher the photon noise. Watson proposed a model of the pupil diameter as a function of age [24] (see also [48]):

(8) $$\begin{array}{c} d\left(\;A\;\right)=D+\left(\;A-28.58\;\right)\times \left(\;0.02132-0.009562D\;\right) \end{array}$$

with *d* and *D* in mm, *A* in years, and

(9) $$\begin{array}{c} D=7.75-5.75\frac{{\left(\text{LSF}\left(n\right)/846\right)}^{0.41}}{{\left(\text{LSF}\left(n\right)/846\right)}^{0.41}+2}. \end{array}$$

*S* is the stimulus area in deg^2^, *L* the adaptation luminance in cd/m^2^, and *n* the condition (*n* = 1: monocular; *n* = 2: binocular). Then, *F*(1) = 0.1 and *F*(2) = 1.

### 4.2. Photon Noise

As stated above, the photon noise describes the statistical fluctuations in the number of incident photons absorbed by the photoreceptors. All photons do not activate a photoreceptor: they can fall between two or reach a photoreceptor without activating it, and missing activating it may increase with age. The photon noise is the inverse of the average flux density of incident photons that cause an activation of the photoreceptors. It depends on the retinal illuminance *E*, on the photon conversion factor *p*, and on the efficiency quantum of the eye *η*, which denotes the rate of activated photoreceptors with respect to the incoming photons:

(10) $$\begin{array}{c} \Phi_{\text{opt}}=\frac{1}{\eta pE}. \end{array}$$

Among these parameters, *E* and *η* both depend on age: the retinal illuminance depends on the pupil diameter *d*, which in turn depends on age [24]. In the photopic domain, the photon conversion factor is set to *p* = 1.285 in the following, as in [17].

#### 4.2.1. Quantum Efficiency

The quantum efficiency is the rate of photons which activate photoreceptors [49]. Van Meeteren measured values as low as 2% [50]. Due to the increasing scattering in ocular media with age, one may expect that the quantum efficiency depends on age. Moreover, *η* may refer to rods or cone quantum efficiency; Barten only takes into account the cone quantum efficiency, which is set to *η* = 3% in his model [17].

Very few studies have associated *η* with age. Hallett has estimated a range of rod quantum efficiency from 0.17 to 0.48% [51], but these results are discussed by Bennett et al. [52]. We found these data too sparse to build a quantitative age-dependent model on it. More data is available about the phototransduction efficiency *F*_1_ [38, 53], which is the ratio between two noises: photon noise in the ocular media and equivalent noise (also called intrinsic noise). For instance, Birch et al. found that *F*_1_ decreases with age and proposed quantitative models of cone and rods phototransduction efficiencies [54]. Unfortunately, it is not possible to estimate *η* from *F*_1_. Finally, due to the lack of available data for the direct estimation of *η*, we estimate this parameter in Section 6, based on available CSF data, as a function of age.

#### 4.2.2. Retinal Illuminance

The retinal illumination depends of the pupil diameter *d* [17]:

(11) $$\begin{array}{c} E\left(\;d\;\right)=\frac{\pi Ld^{2}}{4}\left[\;1-{\left(\;\frac{d}{9.7}\;\right)}^{2}+{\left(\;\frac{d}{12.4}\;\right)}^{4}\;\right] \end{array}$$

with *d* in mm and *L* in cd/m^2^. Thus, *E* depends on age through *d*(*A*), which follows (8).

### 4.3. Neural MTF

The neural MTF describes the signal processing in the visual pathways. It impacts the perception of low spatial frequencies and strongly involves the ganglion cells activity. The receptive field of these cells is known to include a center and a peripheral part, one excitatory and one inhibitive of the input signal [55]. Visual stimuli are transmitted from photoreceptors to these ganglion cells, and the NMTF mainly represents the lateral inhibition due to this excitatory/inhibitive neural architecture of the retina [56]. The main parameter is the drop-off frequency *u*_inh_, which indicates the size of stimuli that can be included in the ganglion cells receptive field. *M*_lat_ depends on *u*_inh_ according to

(12) $$\begin{array}{c} M_{\text{lat}}\left(\;u,e\;\right)=1-e^{-{\left[u/u_{\text{inh}}\left(e\right)\right]}^{2}}. \end{array}$$

The receptive field size of ganglion cells does not seem to increase with age [57]. Thus, we follow Barten's model and consider the drop-off frequency as constant: *u*_inh_(0) = 7 cpd in foveal vision. However, *u*_inh_ depends on the ganglion cells density *N*_*g*_, which in turn depends on age in peripheral vision. This results in the following equation, modified from [17]:

(13) uinhe=uinh0·NgeNg00.851+e/42+0.131+e/202+0.02−0.5.

### 4.4. Neural Noise

Pelli defines the intrinsic noise Φ_eq_ as the sum of the photon noise Φ_opt_ and the neural noise Φ_neu_ [52, 53, 58]. The neural noise corresponds to noise in the visual pathways, between the photoreceptors and the visual cortex. It does not depend on retinal illuminance.

The neural noise seems to be quite age-independent [52, 59, 60]. In the following, we consider it as constant in the fovea, as in Barten's model. However, outside the fovea, we follow Barten and compute Φ_neu_ as a function of the ganglion cells density, which depend on both age and eccentricity. We can write

(14) $$\begin{array}{c} \Phi_{\text{neu}}\left(\;A,e\;\right)=\Phi_{\text{neu}}\left(\;A,0\;\right)\frac{N_{g}\left(A,0\right)}{N_{g}\left(A,e\right)}. \end{array}$$

Neural noise may increase in mesopic conditions; however no data was available in this respect, so that we considered Φ_neu_(*A*, 0) = 3 · 10^−8^ sec·deg^2^ in the fovea whatever the age.

## 5. Cells Densities

The previous section focused on which parameters are needed if one wishes an age-dependent CSF model. In this section, analytical models will be proposed for the cone and ganglion cells densities, as functions of age and eccentricity, using data from the vision science literature.

### 5.1. Ganglion Cells Density

Three types of ganglion cells are present in the retina: P-cells, M-cells, and K-cells, which correspond to the Magno-, Parvo-, and Konio-cellular pathways. To address contrast sensitivity, the relevant cells are the M-cells, responsible for luminance information processing and thus for contrasts sensitivity.

Gao and Hollyfield measured ganglion cells densities for 2 eccentricities (3.5° and 45°) and for various age ranges [46]; ganglion cells densities for different age ranges are also available in [44, 61]. We follow Wassle et al. who estimate that each ganglion cell is roughly connected to three cones whatever the age and eccentricity, so that *N*_*g*_ = 3*N*_*c*_ [61]. From these data and from Barten's model of ganglion cells density (built from observers younger than 37 years old [62]), we have modeled *N*_*g*_ from the foveal cone density *N*_*c*_(*A*, *e* = 0):

(15) NgA,e=3NcA,00.851+e/aA2+0.151+e/7.32,

where

(16) $$\begin{array}{c} a\left(\;A\;\right)=-0.404e^{-0.01246A}-0.1792e^{0.01525A}. \end{array}$$

**Figure 1:**
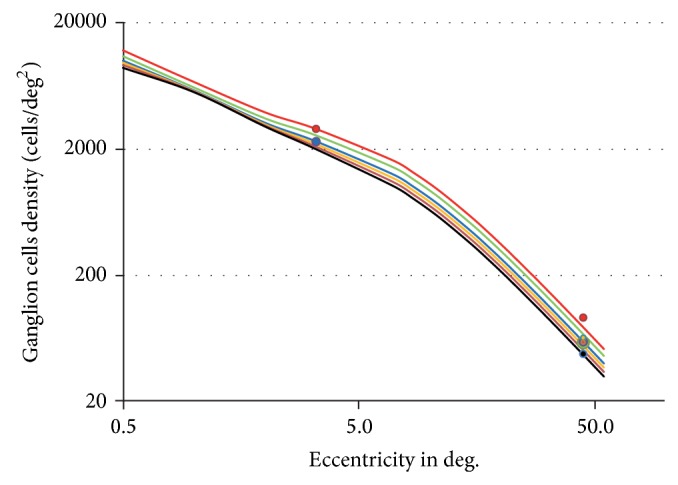
Ganglion cells density as a function of eccentricity and age. Solid curves represent the proposed model (15), and circles are taken from experimental data [44, 46]. Red: 20 years; green: 40 years; blue: 60 years; yellow: 70 years; brown: 80 years; black: 90 years.

This model is plotted in Figure 1 and compared to experimental data [44, 46] for various ages, showing a good agreement.

### 5.2. Cone Density

The cone density is quite stable through ageing, except in the fovea, where it decreases with age [44]. This may contribute to the decline of contrast sensitivity for older observers at high spatial frequencies, where the fovea is required. Gao and Hollyfield conducted experiments for two eccentricities (in the fovea and at *e* = 45°) for observers from 20 to 90 years old [46]. However, the eccentricity was not accurately reported in their paper; moreover, at the center of the fovea, they found a cone density very different to what is found by Curcio et al. [44] and in more recent studies [63, 64].

**Figure 2:**
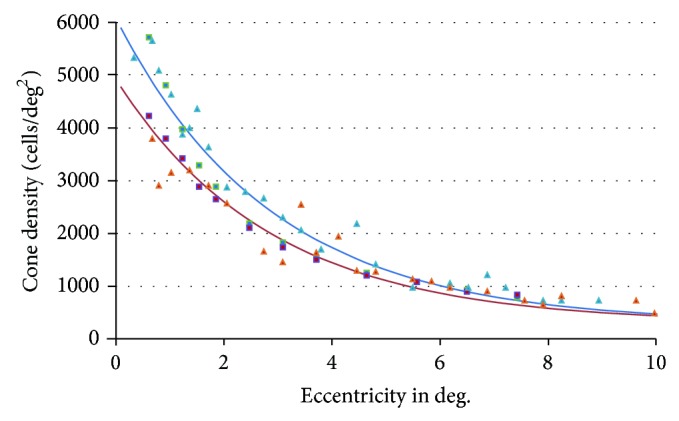
Cone density plotted from experimental data from [63] (blue: 22–35 years old; red: 50–65 years old) and [64] (blue: mean age 27.2; red: mean age 67.2) at various eccentricities. The continuous lines show the model proposed in (17) for 30 years (in blue) and 60 years (in red).

In order to build a quantitative model of *N*_*c*_ as a function of both age and eccentricity, we have used recent data from Song et al. [63] and Chui et al. [64], where eccentricity is explicitly reported. We also used Gao's data for *e* = 45°, as well as Curcio's data [44] (see Figure 2). The cone density is estimated as

(17) $$\begin{array}{c} N_{c}\left(\;A,e\;\right)=\left(\;6952.7-38.70A\;\right)e^{-0.35e}+300. \end{array}$$

The cone density in the fovea is different from Barten's model, which was based on experimental data, where the observer's age was not reported [67–69].

## 6. Parameters Estimation

Due to the lack of direct experimental data, we have estimated four parameters (*η*, *σ*_0_, *u*_opt_, and *k*) as age-dependent functions, by fitting contrast sensitivity data from the vision science literature. To this end, several data sets were considered. We have restricted our investigations to experiments where the ocular pathologies were controlled, because we felt it especially important when dealing with the effects of ageing. In these experiments, the adaptation luminance ranges from 12.5 to 300 cd/m^2^, which is always in the photopic domain. Both monocular and binocular conditions have been considered, for age groups ranging from 20 to 90 years. *T*_*e*_ was set to 0.1 sec, while *u*_0_ = 7 cpd and Φ_0_ = 3 · 10^−8^ sec·deg^2^.

Participants in Owsley's experiment were aged from 20 to 77 years [65]. They were split into three groups with mean ages of 30, 50, and 70 years (*N* = 94). The adaptation luminance was set to 100 cd/m^2^, with 5.5° of field of view. The data was recorded in binocular vision. In Tulunay-Keesey's experiment, the 63 observers were aged from 10 to 70 years [66]. They were split into six groups with mean ages of 17, 25, 34, 45, 54, and 62 years. The adaptation luminance was 60 cd/m^2^, with 7.0° of field of view. The data was recorded monocular vision. In Elliott's experiment, the 24 observers were split into two groups with mean ages of 23 and 69 years [21]. The adaptation luminance was 300 cd/m^2^, with 7.0° of field of view, and data was recorded in monocular vision.

In order to estimate the best parameter values according to these data, we used a Lagrangian optimization, implemented in Matlab. It is a classical applied mathematics method, which allows finding the best set of parameters when considering a given data set (error minimization). Parameters values are tested with a given range and sampling, which were chosen according to the values found in the vision science literature:

- *η* ∈ [0.005; 0.150] (sampling: 0.01%);
- *σ*_0_ ∈ [0.01; 2.00] (sampling: 0.01 arc min/mm);
- *u*_opt_ ∈ [1; 100] (sampling: 2.5 cpd);
- *k* ∈ [0.5; 20.0] (sampling: 0.25).

The optimization led to optimal parameters for each data set: the optimal parameter values are presented in Table 1.

**Figure 3:**
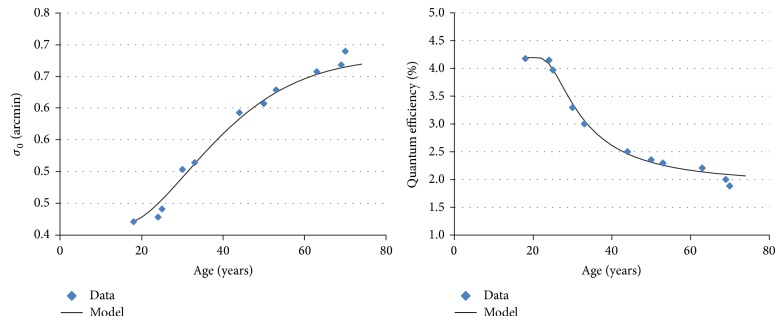
Modeling of *σ*_0_ and *η*, using CSF data from Owsley et al., Tulunay-Keesey et al., and Elliott et al. [21, 65, 66].

The optimal values for *σ*_0_ and *η* are presented in Figure 3. They are consistent with previous findings: first, the standard deviation in ocular media increases with age (Figure 3, left), and the order of magnitude is consistent with Bennett et al. estimates [52]. Second, *η* decreases with age, meaning that less photoreceptors are excited in an old observer's retina, for a given level of photons entering the eye. An analytical model could be fitted to these estimates (solid curves in Table 1):

(18) σ0A0.42+0.26×1−e−A−17/27.18371.547,

(19) ηA=0.019+0.023×1−e−A−17/13.0645−1.753.

**Figure 4:**
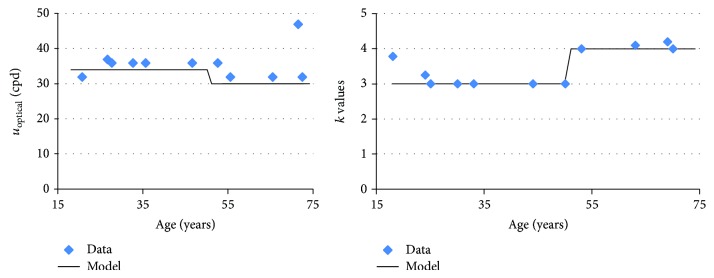
Estimates of *u*_opt_ and *k* as a function of age, using CSF data from Owsley et al., Tulunay-Keesey et al., and Elliott et al. [21, 65, 66].

Figure 4 shows the optimal values of *k* and *u*_opt_. For both parameters, the values are roughly constant below and above 50 years, and a step appears between 50 and 55 years. Thus, we propose choosing *k*(*A*) and *u*_opt_(*A*) as piecewise constant functions:

(20) A50⟹kA=3,uoptA35,

(21) A50⟹kA=4,uoptA30.

## 7. Age-Dependent CSF Model

The proposed age-dependent CSF model generalizes Barten's model:

(22) CSFA,u=MoptA,u2kA·XYTΦoptA,u+ΦneuA,u/MlatA,u.

*M*_opt_(*A*, *u*) is computed from (6), in which *u*_opt_(*A*) follows the above piecewise model (21), along with *k*(*A*). *M*_opt_ also needs an estimate of *σ*_opt_, which in turns needs 3 parameters values (7): the pupil diameter is estimated as a function of age from Watson's formula (8), *C*_*ab*_ is set constant as in Barten's model, and *σ*_0_ is taken from (18).

The optic noise Φ_opt_(*A*, *u*) is computed according to (10) from the retinal illumination *E* (11) and from *η* (19). The neural noise Φ_neu_(*A*, *u*) follows (14), which needs the ganglion cells density *N*_*g*_, available from (15), also using the cone density (17). Finally the lateral inhibition term *M*_lat_(*A*, *u*) follows (12) and needs a model of *u*_inh_, which is available from (13).

**Figure 5:**
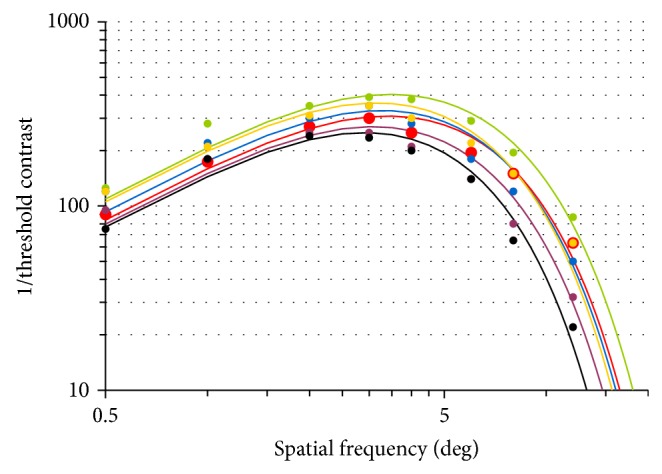
Modeling of Tulunay-Keesey et al.'s data with the proposed age-dependent CSF model (22). Green: 20 years; yellow: 40 years; blue: 60 years; red: 70 years; purple: 80 years; black: 90 years.

In order to test the model's consistency, we have modeled the CSF from the above-cited experiments [21, 65, 66], using (22). For instance, Figure 5 shows the fitting of our model for Tulunay-Keesey's CSF data, at various ages [66].

The Root Mean Square Error (RMSE) was selected as a quantitative estimate of the model's quality, with respect to the data. We have compared this RMSE for the CSF by Owsley, Tulunay-Keesey, and Elliott, either computed with Barten's model or with the proposed model.

When comparing the RMSE for Owsley's CSF data, Barten's model is slightly better for 30-year-old observers (RMSE = 12.54, versus 14.15 with our model), and the proposed model is better for 50-year-old observers (RMSE = 18.82 with Barten's model versus 13.42 with our model) and for 70-year-old observers (45.49 with Barten's model versus 16.42 with our model). Considering now Tulunay-Keesey et al.'s data, Barten's model is slightly better for 17-year-old observers (RMSE = 33.37, versus 36.95 with our model), and our model is better for all other age classes: RMSE = 73.71 with Barten versus 24.69 with our model for 25-year-old observers, 69.46 versus 33.5 for age = 34, 49.99 versus 34.90 with our model for age = 45, 111.90 versus 27.37 for age = 54, and 120.3 versus 19.79 with our model for age = 62. Finally, the RMSE is better with our model for both age classes for Elliott's data: for 23-year-old observers, RMSE = 50.09 with Barten's model versus 40.66 with our model and 104.1 with Barten's model versus 46.45 with our model for 69-year-old observers.

Interestingly, the two CSF where Barten's model is above the proposed model are for 30-year-old and 25-year-old observers, which is in the range of data Barten used to fit his model.

## 8. Conclusion

We have proposed an age-dependent formula to compute the CSF as a function of the spatial frequency. This formula was based on a previous model from Barten [17], which was age-independent. The main reason why we used this model as a starting point was the fact that its parameters are meaningful in a physical or biological sense. Thus, it is possible (at least theoretically) to build an age-dependent CSF model on it from age-dependent models of these parameters. This is what we have done, using published models when available (e.g., Watson's model of the pupil diameter [24]), building models from published data when available (e.g., for the ganglion cells density, see (15)), or estimating an age-dependent model of the parameters from published CSF data, when the first two methods failed.

The proposed formula outperforms Barten's age-independent formula in most cases when applied to the available data, and the difference increases with the observer's age. It is not surprising, given that our new model is fitted to age-dependent data.

To our sense, one of the main results from this study is a negative one: lack of available data. One of the main directions for future research should be to collect experimental CSF data in the mesopic and scotopic domain, in order to extend age-dependent CSF models towards low luminance levels. This lack of age-dependent data also addresses optical parameters, such as the standard deviation *σ*_0_, the cut-off frequency *u*_opt_, and the quantum efficiency *η*. We have proposed quantitative models of these parameters in (18), (19), and (21), but these models were not based on direct measurements of these parameters: they were based on CSF data and need to be improved thanks to direct measurements of the physiological parameters.

Another direction for future research would involve time-dependent stimuli. The temporal CSF of older people is close to the one of younger people for low temporal frequencies, when the stimulus moves slowly. But at high temporal frequency, the performance of older people decreases dramatically [70]. The main difficulty for elderly people is to identify the direction of the stimulus [71, 72]. A model based on monkey studies showed that internal noise increases with age due to a reduced selectivity with respect to the stimulus direction and orientation [41]. Additionally, moving stimuli need more attentional resource, which may also contribute to the impaired performance of elderly people, especially with secondary tasks [73].

Up to now, we have considered the CSF in the spatial domain. In his book, Barten also proposes a formula for the spatiotemporal CSF [17]:

(23) CSFu,w=Moptu2k·XYTΦoptu+Φneuu/H1w1−H2w1−Mlatu,

where *w* is the temporal frequency of the stimuli. *H*_1_ and *H*_2_ correspond, respectively, to the temporal optical MTF (the temporal filtering of the signal captured by photoreceptors) and the temporal component of the lateral inhibition.

A first step towards an age-dependent temporal CSF would be to use (23) with static parameters from our model and *H*_1_ and *H*_2_ as age-independent parameters, which is supported by [74].

Contrast sensitivity decreased at high temporal frequencies whatever the observer's age, so that one can guess that the fitting parameter *k* depends on age and temporal frequency. Also, the optical cut-off frequency *u*_opt_ should depend on these same two factors, given that the optical MTF dramatically decreases at high temporal frequency. Assuming that the optical cut-off frequency *u*_opt_ and *k* both may depend on age and temporal frequency, it is possible to use the age-dependent model proposed in this paper and to estimate *u*_opt_ and *k* from published spatiotemporal CSF data, as we did for spatial frequency.

Appropriate data can be found; for instance, Sloane et al. recruited two groups of observers with mean ages of 23 and 74 years [19]. Sine grating was displayed with a visual angle of 6° in monocular vision, with two temporal frequencies (0.5 and 7.5 Hz) and 8 adaptation luminance values (from 107 to 0.034 cd/m^2^). Data from Tulunay-Keesey et al. is also relevant here [66]: in the abovementioned paper, these authors also considered stimuli with temporal frequencies of 1, 5, and 15 Hz; Elliott et al. also proposed temporal CSF at 4 and 16 Hz [21].

For instance, we have fitted *k* with the experimental data [19, 21, 66], assuming that the dependence on age and temporal frequency are independent of each other:

(24) $$\begin{array}{c} k\left(\;A,w\;\right)=k\left(\;A\;\right)k^{⋆}\left(\;w\;\right). \end{array}$$

**Figure 6:**
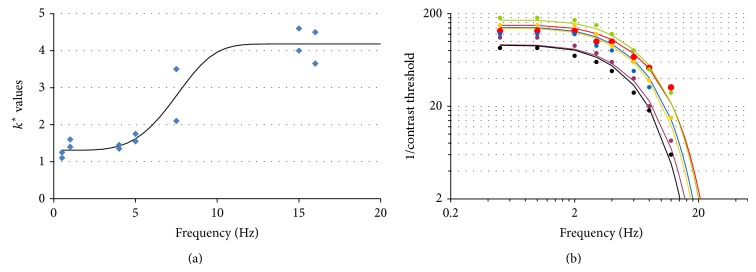
(a) Estimates of *k* ^⋆^ as a function of the stimulus temporal frequency *w*, from experimental CSF data from Elliott, Sloane, and Tulunay-Keesey [19, 21, 66]. (b) Example of the application of the proposed model to age-dependent spatiotemporal CSF from Tulunay-Keesey [66]. Red: 17 years; green: 25 years; blue: 34 years, yellow: 45 years; purple: 54 years; black: 62 years.

Figure 6(a) shows the dependence of *k* ^⋆^ with respect to *w*. It can be expressed as

(25) $$\begin{array}{c} k^{⋆}\left(\;w\;\right)=1.0835+3.1045\left[\;1-e^{-{\left(\left(w-0.5\right)/8.4785\right)}^{2.497}}\;\right]. \end{array}$$

Similarly, an estimation of *u*_opt_ on the same experimental data suggested that this parameter may be considered independent of *w*. An example of the temporal CSF computed with the resulting model is available in Figure 6(b), for data taken from [66].

In this paper, a continuous age-dependent model of CSF has been built upon available CSF data at different ages. Based on this model, it is now possible to envisage some novel adaptive applications, which are able to take into account the age, typically for display applications or lighting applications. This is of utmost importance in an ageing society, where the challenge is that elderly people live longer but also in better conditions.

## Figures and Tables

**Table 1:** Estimates of the CSF model's parameters. Age is in years, *L* in cd/m^2^, *u*_opt_ in cpd, *σ*_0_ in arcmin, and *η* in %.

Authors	Age	*k*	*u*_opt_	*σ*_0_	*η*
Owsley and Sloane (1987) [65]	20–39	3.00	30	0.50	0.0330
—	40–59	3.00	30	0.60	0.0235
—	60–77	4.00	30	0.68	0.0190
Tulunay-Keesey et al. (1988) [66]	17	3.77	30	0.42	0.0420
—	25	3.00	34	0.44	0.0400
—	34	3.00	30	0.51	0.0300
—	45	3.00	34	0.59	0.0250
—	54	4.00	30	0.62	0.0230
—	62	4.10	27	0.65	0.0220
Elliott et al. (1990) [21]	23	3.25	35	0.43	0.0415
—	69	4.2	45	0.66	0.0200
